# Barriers, facilitators and views about next steps to implementing supports for evidence-informed decision-making in health systems: a qualitative study

**DOI:** 10.1186/s13012-014-0179-8

**Published:** 2014-12-05

**Authors:** Moriah E Ellen, Grégory Léon, Gisèle Bouchard, Mathieu Ouimet, Jeremy M Grimshaw, John N Lavis

**Affiliations:** Jerusalem College of Technology, ᅟ, Jerusalem Israel; Centre for Health Economics and Policy Analysis, McMaster University, 1280 Main Street West, CRL 209, Hamilton, Ontario L8S 4K1 Canada; Department of Clinical Epidemiology and Biostatistics, McMaster University, Hamilton, Canada; Israeli Center for Technology Assessment in Health Care, Gertner Institute, Tel Hashomer, Israel; Department of Political Science and CHUQ Research Center, Université Laval, Quebec City, Canada; Department of Medicine, University of Ottawa, Ottawa, Canada; Clinical Epidemiology Program, Ottawa Hospital Research Institute, Ottawa, Canada; Department of Political Science, McMaster University, Hamilton, Canada; Department of Global Health and Population, Harvard School of Public Health, Boston, USA; McMaster Health Forum, McMaster University, Hamilton, Canada

**Keywords:** Evidence informed decision-making, Knowledge transfer and exchange, Knowledge translation

## Abstract

**Background:**

Mobilizing research evidence for daily decision-making is challenging for health system decision-makers. In a previous qualitative paper, we showed the current mix of supports that Canadian health-care organizations have in place and the ones that are perceived to be helpful to facilitate the use of research evidence in health system decision-making. Factors influencing the implementation of such supports remain poorly described in the literature. Identifying the barriers to and facilitators of different interventions is essential for implementation of effective, context-specific, supports for evidence-informed decision-making (EIDM) in health systems. The purpose of this study was to identify (a) barriers and facilitators to implementing supports for EIDM in Canadian health-care organizations, (b) views about emerging development of supports for EIDM, and (c) views about the priorities to bridge the gaps in the current mix of supports that these organizations have in place.

**Methods:**

This qualitative study was conducted in three types of health-care organizations (regional health authorities, hospitals, and primary care practices) in two Canadian provinces (Ontario and Quebec). Fifty-seven in-depth semi-structured telephone interviews were conducted with senior managers, library managers, and knowledge brokers from health-care organizations that have already undertaken strategic initiatives in knowledge translation. The interviews were taped, transcribed, and then analyzed thematically using NVivo 9 qualitative data analysis software.

**Results:**

Limited resources (i.e., money or staff), time constraints, and negative attitudes (or resistance) toward change were the most frequently identified barriers to implementing supports for EIDM. Genuine interest from health system decision-makers, notably their willingness to invest money and resources and to create a knowledge translation culture over time in health-care organizations, was the most frequently identified facilitator to implementing supports for EIDM. The most frequently cited views about emerging development of supports for EIDM were implementing accessible and efficient systems to support the use of research in decision-making (e.g., documentation and reporting tools, communication tools, and decision support tools) and developing and implementing an infrastructure or position where the accountability for encouraging knowledge use lies. The most frequently stated priorities for bridging the gaps in the current mix of supports that these organizations have in place were implementing technical infrastructures to support research use and to ensure access to research evidence and establishing formal or informal ties to researchers and knowledge brokers outside the organization who can assist in EIDM.

**Conclusions:**

These results provide insights on the type of practical implementation imperatives involved in supporting EIDM.

## Background

Research evidence has shown that at times, there is a disconnect between clinical and health services research and health system decision-making [1,2]. Bridging the gap between what we ‘know’ and what we ‘do’ is an important challenge [3]. Limitations in efforts to support the use of research evidence by health system decision-makers (poor communication methods, results not communicated in a timely manner, users not having the appropriate skills) lead to an underutilization of knowledge, which in turn can lead to health system inefficiencies and poor outcomes [4,5]. The term ‘knowledge translation’ (KT) has gained traction as a paradigm to address many of the challenges in translating research to knowledge users and start closing the ‘know-do’ gap [3]. KT is defined as ‘a dynamic and iterative process that includes synthesis, dissemination, exchange, and ethically-sound application of knowledge to improve the health of citizens, provide more effective health services and products, and strengthen the health-care system’ [6].

A plethora of challenges and barriers can be present at various levels within a health system and KT interventions need to address the multiple levels in a health system in order to ensure success [7,8]. For example, ensuring ‘buy-in’ from upper management for the implementation of KT interventions is essential; however, if the appropriate infrastructure is not in place, and managers and directors do not have access to the appropriate research evidence, then KT will be inefficient [9]. Technical barriers (i.e. poor access to research evidence), as well as cognitive barriers (i.e. the lack of knowledge regarding how to identify high quality scientific articles in order to apply only the high-quality research evidence in decision-making), are KT challenges for decision-makers in the health system [10-13]. Due to the range of potential challenges, interventions likely need to be considered that address wider systems issues.

Numerous KT approaches and tools have been developed that are focussed on assisting decision-makers in the health system to increase their use of research evidence in decision-making [14,15]. One framework identified seven main KT approaches to target health system decision-makers: 1) establishing a climate for research use, which includes activities undertaken by the organization and the health system to establish a climate where research evidence is used in decision making (i.e., organizational mission and vision that states the importance of evidence in decision-making), 2) research production efforts, which include activities taken by researchers, funders, and knowledge users, to ensure the production of timely and relevant research (i.e., participating in priority setting processes), 3) ‘push’ efforts, which include activities usually undertaken by researchers or intermediaries (i.e., librarians or knowledge brokers) to disseminate research evidence to potential knowledge users (i.e. using a knowledge intelligence service that scans and distributes relevant research), 4) ‘facilitating pull’ efforts, which focus on activities that the health system needs to undertake in order to ensure that the appropriate infrastructure is in place for knowledge users to access the necessary research evidence (i.e., enabling easy access to research through the intranet), 5) ‘pull’ efforts, which include activities by health system decision-makers to enable the appropriate use of research evidence, 6) ‘linkage and exchange’ efforts, which include activities that focus on facilitating relationships between researchers and knowledge users (i.e., providing training and continuing education related to the use of research in decision making), and 7) evaluation efforts which include evaluations of knowledge translation interventions and outcomes (i.e., monitoring and evaluating knowledge translation initiatives) [5,16]. These seven domains are the main components of a framework that was used to guide the research. In earlier phases of the research, published elsewhere, we identified different supports within each domain that organizations can implement to support EIDM [9,16].

This current paper builds on and complements (a) an environmental scan in Canadian health-care organizations and scoping review of the literature on supports (i.e., positions, programs, interventions, instruments, or tools) implemented across the health systems to support EIDM, (b) the development of a framework that identified the various infrastructural components that an organization or health system can implement to support EIDM, and (c) a qualitative study that showed the current mix of supports that some Canadian health-care organizations have in place to facilitate EIDM [9,16].

While a lot of research is in progress with respect to EIDM, specifically in clinical practice, further innovation and interventions need to be considered to accelerate the uptake and implementation of research evidence at the level of the health system. In order to accelerate the changes, it is essential to understand the barriers and facilitators and present the current thinking as to what the future priorities should be focused on in order to enhance EIDM in the health system. The purpose of this paper is to identify (a) barriers and facilitators to implementing supports for EIDM in Canadian health-care organizations, (b) views about emerging development of supports for EIDM, and (c) views about the priorities to bridge the gaps in the current mix of supports these organizations have in place.

## Methods

This study addresses unpublished data from in-depth, semi-structured telephone interviews that were conducted in three types of health system organizations: regional health authorities (RHAs), hospitals, and primary care practices (PCPs) in two Canadian provinces (Ontario and Quebec) as part of a previous qualitative study. For rationale on the study design, or for further details on the methodology, please see Ellen [9].

### Developing the interview guide

An environmental scan and scoping review that identified the potential supports that an organization or health system can have in place for EIDM served as the basis for the interview guide [16]. We defined these supports as any instrument or intervention (i.e., positions, programs, interventions, instruments, or tools) implemented in health-care organizations or broader health systems of which they are a part in order to facilitate access to and dissemination, exchange, and/or use of research evidence. The seven main domains of supports that were identified were: i) climate for research use, ii) research production, iii) push efforts, iv) facilitating pull efforts, v) pull efforts, vi) linkage and exchange efforts, and vii) evaluation efforts [5,16]. We developed an interview guide using the seven main domains for guiding the responses to the questions. The focus of the interviews was to explore (a) views about the most important supports for EIDM in their organization (results of which are available in [9]), (b) barriers and facilitators to implementing these supports, (c) views about emerging development of supports for EIDM, and (d) views about the priorities to bridge the gaps in the current mix of supports their organization have in place. Eight overarching questions were developed that addressed the four main components of the interview. The interviews were specifically focused on the organizational supports that are needed to implement EIDM. We informed participants that when we use the word evidence, we refer to academic research outputs (i.e., articles, research reports, and books) and population and health system data (i.e., surveillance data, service utilization data, and other non-financial performance data).

### Selecting and recruiting the sample

The sample was drawn at the provincial, organizational, and employee level. This three-stage procedure facilitated the identification of the appropriate individuals in the organizations that have already implemented a number of KT interventions [9]. We purposely wanted to obtain the insights from ‘high-performers’ , on the type of supports that could be developed for EIDM in the health system. By ‘high-performers’ , we refer to organizations that are ‘high-performers’ within the field of KT i.e., these organizations have already invested and demonstrated a commitment to KT at the organizational level, such as participation in the Executive Training for Research Application (EXTRA) program from the Canadian Health Services Research Foundation, now called the Canadian Foundation for Healthcare Improvement.

At the first stage, we purposively selected to conduct the interviews in the two largest Canadian provinces (Ontario and Quebec). These two Canadian provinces have invested in KT initiatives and have significant experience in the field. At the second stage, we selected three types of organizations in the health system (RHAs, hospitals, and PCPs) because they are accountable for the funding and/or the delivery of the bulk of health-care services in the two provinces. We sampled organizations that have already participated in strategic behavior with respect to KT activities, since as was mentioned before, we want to learn from the ‘high performers’. For further details, please see the study protocol [16]. We chose three RHAs, five to six hospitals, and six PCPs in each province. At the last stage, we purposely sampled positions. Our goal was to interview individuals in three different types of positions (i.e., a senior manager, such as a Chief Executive Officer, Chief Nursing Officer, or Vice President of Quality Improvement, who will be more focused on the organizational infrastructure; a library or resource center manager, who will be more focused on the technical infrastructure; and a knowledge broker or someone in a position that implies supporting evidence-informed decision-making in the organization, management and, delivery of health services) in each organization that could provide us with a broad view, as well as different perspectives, on the implementation of supports for EIDM in their organization. We also used the chain-referral sampling technique by asking the contacted participants to identify other individuals within the organization who, in their view, were most appropriate to participate in the interview [17].

Due to the fact that not all organizations would have all three position types, we strived to interview at least one, but ideally three, participants in each organization. We attempted to conduct, at most, 18 interviews in RHAs (3 interviews in 3 RHAs each in Ontario and Quebec), 30 in hospitals (3 interviews in 5 hospitals each in Ontario and Quebec), and 24 in PCPs (3 interviews in 4 PCPs each in Ontario and Quebec), for a total potential of 72 interviews. A cover letter, consent form, and project summary were sent to each potential participant. Follow-up emails and phone calls were made, when necessary. Each individual who chose to take part in our study participated in an in-depth semi-structured interview, often surpassing our 45-min length guideline. The median length of interview was 50 min.

### Data analysis

Interviews were audio-recorded, transcribed, de-identified, checked for accuracy, and then analyzed thematically. Field notes were kept during the telephone interviews and were utilized during the data analysis. NVivo 9 (QSR International, Cambridge, MA), a software package for qualitative data analysis, was used for data management and coding. First and second level themes were developed based on the framework that was developed during the scoping review [16]. Third-level themes were developed inductively by ME, GL, and GB throughout the analysis and were based on themes that were recurrent in the semi-structured interviews. A constant comparative method was used for the thematic analysis of the data.

Interviews were analyzed in clusters. First, the entire interview was read to get a sense of the whole interview and initial impressions. Second, while reading through the interview a second time, we coded units of text into nodes and subnodes and compared initial codes between coders. Three researchers (ME, GL, and GB) conducted the coding and comparison. At least two researchers coded each interview, and, together with the project team, we revisited the overall coding framework and revised it until agreement was reached.

For each coded element, we calculated the frequencies and percentages of total number of participants by organization, province, and position type. Only elements that were coded for ≥10% of the total number of participants were deemed of sufficient frequency for inclusion. Next, we searched for differences in coded responses that were >50% of the total number of participants within organization, province, and position type. However, no such differences in coded responses were found within organization, province, and position type.

The following frequency taxonomy is used to describe the results: ‘all’ refers to 100%, ‘most’ refers to 67% to 99%, ‘many’ refers to 33% to 66%, ‘some’ refers to 1% to 32%, and ‘none’ refers to 0% of the total participants. Considering that none of the sub-elements were mentioned by ‘all’ or ‘most’ of the participants, an effort was made in the results section to include only sub-elements that were mentioned by ‘many’ (i.e., between 33% to 66%) of the participants. However, in some instances, none of the sub-elements were mentioned by ‘many’ of the participants, and therefore, sub-elements that were mentioned by ‘some’ (i.e., between 1% to 32%) of the participants were included.

### Ethics

The study protocol was submitted to and approved by the Hamilton Health Science and the CHU de Québec Research Ethics Boards, for Ontario and Quebec, respectively.

## Results

One hundred and four invitations were sent by email with the goal of having a total of 72 respondents; 27 were non-responders and 20 declined. Fifty-seven interviews were conducted in 24 organizations (i.e., 6 RHAs, 11 hospitals, and 7 PCPs) in Ontario and Quebec (for more details, see Table 1, reproduced from [9]).

**Table 1:** **Interview participants by organization and position type**

**Province**	**Number of interview participants (% of total)**						
	**Total**	**Organization**			**Position**		
		**PCP** ^**a**^ **(7 organizations)**	**Hospital (11 organizations)**	**RHA** ^**b**^ **(6 organizations)**	**Senior manager**	**Knowledge broker**	**Library manager**
Ontario	29 (51%)	3 (5%)	14 (25%)	8 (14%)	19 (33%)	5 (9%)	5 (9%)
Quebec	28 (49%)	6 (11%)	18 (32%)	8 (14%)	14 (25%)	6 (11%)	8 (14%)
Total	57 (100%)	9 (16%)	32 (56%)	16 (28%)	33 (58%)	11 (20%)	13 (23%)

^a^ *PCP* primary care practices.

^b^ *RHA* regional health authority.

### Barriers and facilitators to implementing supports for EIDM

Most participants reported one or more barriers to implementing supports for EIDM in their organizations. Based on frequency of responses, the three most frequently reported barriers were limited resources (money or staff), time constraints, and negative attitudes toward change (Table 2).

**Table 2:** **Barriers and facilitators to implementing supports for EIDM**

	**Number of interview participants (** ***n*** **)** ^**a**^								
	**Total (n = 57)**	**Organization** ^**b**^			**Province**		**Occupation**		
		**PCP (** ***n*** **= 9)**	**Hospital (** ***n*** **= 32)**	**RHA (** ***n*** **= 16)**	**Ontario (** ***n*** **= 29)**	**Quebec (** ***n*** **= 28)**	**Senior manager (** ***n*** **= 33)**	**Knowledge broker (** ***n*** **=11)**	**Library manager (** ***n*** **= 13)**
**Barriers**	***50***	***8***	***29***	***13***	***24***	***26***	***28***	***10***	***12***
-Limited resources^c^	34	***6***	19	9	16	18	19	***8***	7
-Time constraints	23	***6***	11	6	16	*7*	12	4	7
-Negative attitude toward change	*14*	3	*8*	*3*	*7*	*7*	*7*	4	*3*
**Facilitators**	30	4	18	8	17	13	18	***8***	*4*
-Genuine interest from health system decision-makers	23	3	15	*5*	16	*7*	14	7	*2*

^a^Percentage of total number of participants (font style used): 0% of participants (bold); 1% to 32% of participants (italic); 33% to 66% of participants (regular i.e. not bold and not italic); 67% to 99% of participants (bold and italic); 100% of participants (regular and underlined).

^b^ *PCP* primary care practice, *RHA* regional health authority.

^c^The main two sub-elements that were mentioned within the category of ‘limited resources’ were: money (*n* = 19) and staff (*n* = 14).

Many participants stated that their organizations had insufficient resources (i.e. money) to support organizational or technology infrastructures, training, or capacity building related to supporting EIDM. One participant stated ‘I think the biggest barrier is just resources. The hospital has a very limited set of resources that it can bring to bear on this because it can’t use ministry money to support non-ministry activities’. The lack of resources is especially felt in the libraries, as is demonstrated by this participant’s comment; ‘currently, the library services really need a lot of technology and cutting-edge technologies. At the same time, it is often a service where we begin to realize its added value, but we still tend not to increase the budget (…) It is often the “poor relative” and unfortunately it should not be that way’.

Some participants stated that frequent staff turnover is a barrier to EIDM. When the champions of EIDM leave the organization, ‘it’s sort of like starting over again’. For example, ‘There was a senior director here who had research articles that she would gather the team together to discuss. Please read this article, here are three questions, let’s meet and discuss. This person no longer is with us at the [RHA] and since her departure; we have not picked that back up. (…) Why nobody else picked it up, I don’t know’.

Many participants stated that they have limited time to make decisions or limited free time for training related to EIDM; ‘The biggest limitation on decision-makers in a hospital like this one anyway, is that you don’t have a whole heck of a lot of time to pause and look for the evidence. It’s a really serious problem (…) It’s more than just getting someone to do a literature review. It’s about having a discussion and considering the evidence’. Even in situations where the organization was committed to freeing up time of its senior staff to attend training programs and run with EIDM projects, the time was rarely available; ‘you were to have 20% of your time freed up to be able to work on your project and your EXTRA [Executive Training for Research Application program from the Canadian Foundation for Healthcare Improvement, formerly the Canadian Health Services Research Foundation] Fellowship. To be honest, most of us have ended up doing it on weekends because of the intensity’.

Some participants stated that there is a negative attitude (or resistance) toward change; ‘…hospitals are intrinsically resistant to change because they do things pretty well now and so they aren’t all that interested in changing the way they do things’. Furthermore, some staff had a negative reaction to the use of evidence and KT; ‘there is so much funded garbage going on in research in academia and elsewhere. Anyone who looks at [national funding body] and some of the things that have been funded and the lack of direct applicability, I think for people who aren’t researchers and don’t live in academia, it just looks like so much garbage. And now we have people on the user end of it saying you know what, there are more questions than there are answers and all the so-called evidence that has been shoved down our throats for so long wasn’t really evidence (…)’.

The most frequently reported facilitator to implementing supports for EIDM was a genuine interest from health system decision-makers, notably their willingness to invest money and resources and to create a KT culture over time in health-care organizations. The CEO’s push and drive to ensure EIDM was a strong force in ensuring the use of research evidence; ‘People will copy the leader and if the leader is pushing a type of behavior, then that will be pushed in the organization. So if the leader values research and that’s shown by either putting money toward it or by asking people to justify their choice of action based on data, then that will propagate through the organization’. Furthermore, having the support from senior management was also viewed as essential; ‘Well, the facilitation was having a few dedicated staff people (…) very strong and fundamental support from senior management that they knew that all of senior management was very much on-side which helped promote the project’ , and ‘The senior management push, I would say that probably was introduced because of a couple of individuals in senior management who just said this is really important and you really need to go this way. So we’ve had a couple of senior VPs who really have been the push to say you need to use evidence and that’s either because they’ve come from a clinical background and want to see it done at a management level or just because they’ve had the education themselves. Some of them are PhDs who see the value’.

### Views about emerging development of supports for EIDM

Participants were asked to identify initiatives that were emerging (i.e., coming ‘down the pipe’) in their organization or in the broader health-care system that they thought offer promise to support EIDM. Many participants stated that facilitating pull efforts, notably implementing accessible and efficient systems to support the use of research in decision-making (e.g., documentation and reporting tools, communication tools, and decision support tools), were the focus of initiatives that were emerging to support EIDM (Table 3). Participants referred to new databases, business intelligence tools, and decision support tools that will enable EIDM; ‘A business intelligence tool is in development by the Ministry of Health’, and ‘The integrated decision support tool that is housed at [consortium of hospital] has really grown and continues to grow. In terms of the beta holdings that it has acquired and produces reports on and allows us to make kind of evidence-based decisions on’.

**Table 3:** **Views about emerging development of supports for EIDM**

**Support for evidence-informed decision-making**	**Number of interview participants (** ***n*** **)** ^**a**^								
	**Total (** ***n*** **= 57)**	**Organization** ^**b**^			**Province**		**Occupation**		
		**PCP (** ***n*** **= 9)**	**Hospital (** ***n*** **= 32)**	**RHA (** ***n*** **= 16)**	**Ontario (** ***n*** **= 29)**	**Quebec (** ***n*** **= 28)**	**Senior manager (** ***n*** **= 33)**	**Knowledge broker (** ***n*** **= 11)**	**Library manager (** ***n*** **= 13)**
**Facilitating pull efforts**	25	4	14	7	*8*	17	11	6	8
-Implement accessible and efficient systems to support the use of research in decision-making^c^	*14*	4	*6*	*4*	*6*	*8*	*9*	*2*	*3*
-Implement technical infrastructure to support research use and to ensure no restrictions are placed on staff’s access to online resources that contain relevant research evidence	*12*	*1*	*10*	*1*	*4*	*8*	*3*	*3*	6
-Provide easy access to journals and scientific literature either through bulk purchasing of subscriptions or promoting open-access resources^d^	*10*	*1*	*6*	*3*	*2*	*8*	*4*	*1*	5
**Climate for research use**	19	*2*	14	*3*	*8*	11	13	*3*	*3*
-Develop and implement an infrastructure or positions where the accountability for encouraging knowledge use lies^e^	*13*	**0**	*10*	*3*	*5*	*8*	*7*	*3*	*3*
Pull efforts	*16*	*2*	*10*	*4*	*8*	*8*	*7*	7	*2*
-Enable training and continuing education that focus on finding and using research evidence in decision-making	*8*	*1*	*6*	*1*	*3*	*5*	*5*	*1*	*2*
-Ensure decision-making processes promote the use of research in decision-making	*7*	*1*	*5*	*1*	*4*	*3*	*3*	*3*	*1*
**Linkage and exchange efforts**	*14*	3	*7*	*4*	*6*	*8*	*8*	*2*	*4*
-Establish formal and informal ties to researchers and knowledge brokers outside the organization who can assist in integrating evidence into decision-making^f^	*13*	3	*6*	*4*	*5*	*8*	*8*	*2*	*3*
**Push efforts**	*14*	**0**	*10*	*4*	*3*	11	*5*	*3*	6
-Use a knowledge intelligence service that scans the literature and distributes research evidence throughout the organization^g^	*11*	**0**	*7*	*4*	*2*	*9*	*4*	*1*	6
**Research production efforts**	*9*	*2*	*6*	*1*	*3*	*6*	*5*	4	**0**
**Evaluation of efforts to link research to action**	*7*	*1*	*3*	*3*	*2*	*5*	*3*	*3*	*1*

^a^Percentage of total number of participants (font style used): 0% of participants (bold); 1% to 32% of participants (italic); 33% to 66% of participants (regular i.e. not bold and not italic); 67% to 99% of participants (bold and italic); 100% of participants (regular and underlined).

^b^ *PCP* primary care practice, *RHA* regional health authority.

^c^The main two sub-elements that were mentioned within the category of ‘Implement accessible and efficient systems to support the use of research in decision-making’ were: decision support tools (*n* = 8) and documentation and reporting tools (*n* = 8).

^d^The main sub-element that was mentioned within the category of ‘Provide easy access to journals and scientific literature’ was: electronic-based resources i.e. bibliographic databases (*n* = 6).

^e^The main sub-element that was mentioned within the category of ‘Develop and implement an infrastructure or positions where the accountability for encouraging knowledge use lies’ was: department or section dedicated to KT (*n* = 5).

^f^The main sub-element that was mentioned within the category of ‘Establish formal and informal ties to researchers and knowledge brokers’ was: being part of group outside the institution (*n* = 9).

^g^The main sub-element that was mentioned within the category of ‘Use a knowledge intelligence…’ was: information monitoring services (*n* = 7).

Many participants also stated that establishing a climate for research use, notably developing and implementing an infrastructure or position where the accountability for encouraging knowledge use lies, was among the priorities for next steps in further development of supports for EIDM. One participant said that this was necessary since ‘people who do health services research, they’re not well connected to the decision makers in the network and so we’re going to consider what options there are to bring that culture together’. Some organizations have already started to formalize the process; ‘We have now an expanded risk and management of quality and insurance department that has its goal in the next couple of years to start bringing research from a broader basis, you know looking at the whole health care system. It’s benchmarking kind of information’s back to our individual missions to help people with quality improvement initiatives’.

### Views about the priorities to bridge the gaps in the current mix of supports for EIDM

Many participants stated that facilitating pull efforts, notably implementing technical infrastructures to support research use and to ensure access to research evidence, were among the priorities to bridge the gaps in the current mix of supports for EIDM (Table 4). Participants discussed the need for either (a) a central repository of knowledge; ‘I think something that we alluded to that I think would be great is to have some kind of centralized repository of knowledge or sharing (…) a really good central database of research output (…) on a local, provincial or even larger level’ , (b) access to journals and databases; ‘we don’t have access to the consortium of libraries (…) Currently, to search the bibliographic databases we use our colleagues’ login, which have a professorial status or the equivalent, to perform such research,’ or (c) appropriate wireless access within the organizations; ‘the lack of wireless technology in hospitals has been a problem for the ease of research in some ways’.

**Table 4:** **Views about priorities to bridge the gaps in the current mix of supports for EIDM**

**Support for evidence-informed decision-making**	**Number of interview participants (** ***n*** **)** ^**a**^								
	**Total (** ***n*** **= 57)**	**Organization** ^**b**^			**Province**		**Occupation**		
		**PCP (** ***n*** **= 9)**	**Hospital (** ***n*** **= 32)**	**RHA (** ***n*** **= 16)**	**Ontario (** ***n*** **= 29)**	**Quebec (** ***n*** **= 28)**	**Senior manager (** ***n*** **= 33)**	**Knowledge broker (** ***n*** **= 11)**	**Library manager (** ***n*** **= 13)**
**Facilitating pull efforts**	29	4	16	9	15	14	14	***8***	7
-Implement technical infrastructure to support research use and to ensure no restrictions are placed on staff’s access to online resources that contain relevant research evidence^c^	*17*	3	*9*	*5*	*6*	11	*8*	4	5
-Provide easy access to journals and scientific literature either through bulk purchasing of subscriptions or promoting open-access resources^d^	*11*	*2*	*7*	*2*	*2*	*9*	*4*	4	*3*
-Implement accessible and efficient systems to support the use of research in decision-making^e^	*10*	*2*	*5*	*3*	*7*	*3*	*7*	*1*	*2*
**Linkage and exchange efforts**	*18*	4	*8*	6	10	*8*	12	4	*2*
-Establish formal and informal ties to researchers and knowledge brokers outside the organization who can assist in integrating evidence into decision-making^f^	*17*	4	*7*	6	*9*	*8*	12	4	*1*
Pull efforts	*18*	3	*10*	*5*	12	*6*	*10*	5	*3*
-Enable training and continuing education that focus on finding and using research evidence in decision-making	*8*	*1*	*7*	**0**	*5*	*3*	*4*	*1*	*3*
-Ensure decision-making processes promote the use of research in decision-making	*6*	*1*	*5*	**0**	*4*	*2*	*4*	*1*	*1*
**Climate for research use**	*16*	*1*	12	*3*	*8*	*8*	11	*3*	*2*
-Develop and implement an infrastructure or positions where the accountability for encouraging knowledge use lies	*11*	*1*	*8*	*2*	*4*	*7*	*7*	*2*	*2*
Evaluation efforts to link research to action	*13*	3	*7*	*3*	*6*	*7*	*7*	*2*	*4*
Push efforts	*12*	*2*	*9*	*1*	*2*	10	*8*	*2*	*2*
-Use a knowledge intelligence service that scans the literature and distributes research evidence throughout the organization^g^	*8*	**0**	*7*	*1*	*2*	*6*	*5*	*2*	*1*
**Research production efforts**	*10*	4	*4*	*2*	*4*	*6*	*7*	*3*	**0**

^a^Percentage of total number of participants (font style used): 0% of participants (bold); 1% to 32% of participants (italic); 33% to 66% of participants (regular i.e. not bold and not italic); 67% to 99% of participants (bold and italic); 100% of participants (regular and underlined).

^b^ *PCP* primary care practice, *RHA* regional health authority.

^c^The main two sub-elements that were mentioned within the category of ‘Implement technical infrastructure’ were: access inequalities (*n* = 6) and restrictions (*n* = 5).

^d^The main two sub-elements that were mentioned within the category of ‘Provide easy access to journals and scientific literature’ were: electronic-based resources i.e. bibliographic databases (*n* = 8) and access through a network i.e. library consortium (*n* = 6).

^e^The main sub-element that was mentioned within the category of ‘Implement accessible and efficient systems’ was: decision support tools (*n* = 7).

^f^The main two sub-elements that were mentioned within the category of ‘Establish formal and informal ties to researchers and knowledge brokers’ were: links to individual researchers, experts or opinion leaders (*n* = 10) and being part of groups outside the institution (*n* = 6).

^g^The main sub-element that was mentioned within the category of ‘Use a knowledge intelligence service’ was: information monitoring services (*n* = 6).

Some participants also stated that linkage and exchange efforts, notably establishing formal or informal ties to researchers and knowledge brokers outside the organization who can assist in EIDM, were among the priorities to bridge the gaps in the current mix of supports for EIDM; ‘Well, ideally for us, it would be some kind of linkage (…). Our formal relationships are topic-specific or issue-specific, versus general. So if I think of some of the EXTRA [Executive Training for Research Application] faculty who have that sort of general interest and expertise in knowledge exchange, I think down the road that people with that kind of expertise would be helpful to organizations like us in building a more formal relationship with them’.

## Discussion

### Summary of study findings

This study identified the barriers, facilitators, and views about the next steps to implementing supports for EIDM in three types of health system organizations (RHAs, hospitals, and PCPs) in two Canadian provinces (Ontario and Quebec). We found three main barriers (i.e., limited resources, time constraints, and negative attitude toward change) and one main facilitator (i.e., genuine interest from health system decision-makers). Three of the seven domains from the aforementioned framework of organizational supports that can support EIDM (i.e., ‘facilitating pull’ efforts, ‘establishing a climate for research use’, and ‘linkage and exchange’ efforts) were repeatedly highlighted when we had asked participants about emerging development of supports and main priorities to fill the gap in the current supports. The next most highlighted domain was ‘pull efforts’. The two main views about emerging development of supports for EIDM fell under the domains of ‘facilitating pull efforts’ i.e., implementing accessible and efficient systems to support the use of research in decision-making, and ‘establishing a climate for research use’ i.e., developing and implementing an infrastructure or position where the accountability for encouraging knowledge use lies. The two main priorities to bridge the gaps in the current mix of supports for EIDM fell under the domains of ‘facilitating pull’ efforts, i.e., implementing technical infrastructures to support research use and to ensure access to research evidence, and ‘linkage and exchange efforts’ i.e., establishing formal or informal ties to researchers and knowledge brokers outside the organization who can assist in EIDM.

### Relation to other studies

The results presented here follow a previous qualitative paper that highlighted the current mix of supports that Canadian health-care organizations have in place and the ones that have proven helpful for EIDM [9]. Altogether, these results provide new insights on the practical implementation imperatives involved in supporting EIDM.

Frameworks exist which provide an overarching view of what needs to be undertaken in order to facilitate the transfer of knowledge into practice and some of the future priorities mentioned in this study are in alignment with these frameworks [18-23]. However, we are unaware of any study that provides insight into the knowledge users’ perspectives on what the future priorities should be in developing an infrastructure to support EIDM in three types of health-care organizations. Although the frameworks are useful in understanding the bigger picture, having insight as to where the gaps are and what supports are viewed as future priorities, is extremely valuable. Implementing ‘facilitating pull efforts’ , such as the appropriate technical infrastructures, are integral in ensuring ‘easy access’ to research through physical tools. ‘Pull efforts’ and ‘linkage and exchange efforts’ ensure that decision-makers have the necessary skills and connections to acquire, assess, adapt, and apply the necessary evidence into their decision-making. Finally, all these initiatives reinforce the overall climate that is needed to support EIDM. While the frameworks identify additional initiatives, based on the results of this study, it seems as if these four are the top priorities that need to be focussed on in the coming years.

The barriers and facilitators that were identified in this research mostly align with those that have been discussed in the KT and change management literature. Introducing new initiatives, ways of working, and decision processes presents many challenges to organizations [13].

With respect to the barriers, any change or new initiative requires significant investment in resources, both financial and human; without the investment in the necessary resources, the change will be difficult to implement. Generally, decision-makers in the health services sector are already overburdened with new legislation and initiatives, and adding additional responsibilities, such as KT initiatives, onto their already overflowing plate is not the solution. Appropriate investment in both human and financial resources will ensure that initiatives to support EIDM have the best chance at success. Furthermore, the change management literature demonstrates that it is human nature to resist change and, therefore, the barrier that was discussed in our research, regarding a general resistance to change, is well documented, but so too are potential strategies to support change [24,25]. The facilitators are also in alignment with current change management and KT literature. The need to ensure ‘buy-in’ from upper management, have highly motivated individuals that lead the change, and build a strong climate for research use are all elements that have been identified in the literature [13,25-27].

### Strengths and limitations

The main strengths of this study are, firstly, that the interview participants were from the three main types of health services organizations that are primarily responsible for delivering the bulk of the services that are provided in Ontario and Quebec and, secondly, that up to four respondents were interviewed in each organization (for a total of 57 interviews), thus increasing our confidence in the presented data. The main limitations of this study are, firstly, that findings cannot be said to be generalizable, as this study focussed on organizations that have already invested and implemented strategic infrastructures to support EIDM. Secondly, participation in the interviews from the PCPs was quite low, which could be due to a lack of human resources responsible for KT activities (i.e., librarians or individuals in a knowledge broker-type position). Thirdly, despite our efforts to limit methodological bias (notably during data collection and analysis), the results are based on qualitative research which is by nature prone to a degree of potential bias and subjectivity.

### Future research

The current study is phase 2 in a larger program of research. The following phase will consist of a cross-sectional survey among all general hospitals and RHAs in Ontario and Quebec to identify which supports are currently in place to support EIDM. This current research, in combination with the third phase, may serve as a springboard to identifying where the actual gaps exist and where targeted interventions are necessary. In this paper, we identified the main priorities in addressing the gaps in the current infrastructure but further work is needed in determining which organizational and/or technology infrastructures are needed in which setting. We are essentially discussing the need for a large-system transformation, which requires further understanding of which initiatives work under which circumstances [25]. Context plays a major role in the effectiveness of any health system change, and understanding the contextual factors associated with the success or failure of any KT intervention is an important next step. Furthermore, taking economies of scale into account, it is not feasible for all initiatives to be undertaken by each organization in the health sector. Further work is needed to determine which of the initiatives can be addressed at the federal, provincial, regional, and organizational levels.

Lastly, very little research is being conducted on the effects of interventions to support EIDM. Research on KT tools and processes that can facilitate EIDM is also needed [28,29]. Currently, evaluation of EIDM is not strong in the organizations we included in our study. Future research should examine KT tools and infrastructural components to identify which elements are successful in which contexts [9].

### Implications

As was previously mentioned, this study is phase 2 in a larger program of research. Altogether, this phase identified what supports are currently in place for EIDM in Canadian health-care organizations, the barriers and facilitators to implementation of these supports, views about emerging development of supports for EIDM, and views about the priorities to bridge the gaps in the current mix of supports. It is well known that the implementation of new ideas, changes to infrastructures, and organizational innovation has historically been a difficult process in health organizations and systems [26,30]. Phase 2 focussed on organizations that have already invested and implemented strategic infrastructures to support EIDM; thus, we can learn from the high performers regarding what they view and experience as barriers and facilitators. Going forward, organizations can benefit by learning from these organizations that have gone through the process of implementing an infrastructure that supports EIDM and build on the facilitators, while attempting to minimize the barriers.

Organizations that want to build a system that supports EIDM should explore what has already been put in place by high performers in this area [9], but also take into account the future priorities presented in this paper and determine if there is strategic alignment between future priorities and the organizations’ strengths.

If EIDM is to be achieved, ways must be found to address the barriers and develop an enhanced institutional infrastructure that can support EIDM. There were three main barriers identified in this research i.e. limited resources (money or staff), time constraints, and negative attitudes toward change. There were three main domains that were identified in the research as emerging supports and future priorities i.e., facilitating pull efforts, establishing a climate for research use, and linkage and exchange efforts. In the previous work, we had identified different initiatives that can be undertaken within each of these domains [9,16]. Some of these previously identified initiatives, as well as other initiatives that have since been published, can address the identified barriers.

With respect to the first barrier i.e., limited resources, this is a barrier that has been identified in other sources, specifically with respect to EIDM. Numerous studies have highlighted the limited organizational capacity to collect and evaluate research to use for EIDM [31-33]. However, different initiatives have been discussed in the literature that can address this barrier. For example, under the domain of ‘establishing a climate for research use’ (one of the main domains highlighted by participants in this research), one of the sub-elements is establishing clear points of contacts within the organization regarding where to turn to obtain research evidence. These contacts can supplement internal capacity with external expertise. Therefore, every unit in an organization is not required to have the necessary skills to acquire, assess, adapt, and apply research evidence; however, clear contacts within the organization can assist in consolidating and streamlining the process. Additionally, one sub-element mentioned by participants that can address this barrier, under the ‘linkage and exchange domain,’ was having formal and informal relationships to people outside the organization who can assist in obtaining the appropriate research evidence. Fostering relationships and collaborating with experts in the field are ways to address this barrier [34,35]. In an Australian study that examined the use of evidence in policy, participants suggested implementing bridging systems between researchers and policy makers and having a standing arrangement with key research groups as ways to facilitate the use of research to inform decision-making [36]. Organizations may not have the financial or human resources to train internal staff to support EIDM; however, links with researchers and academics outside of the organization can help facilitate EIDM.

The second most common cited barrier was time constraints. Health system employees are already overburdened with effectively executing their jobs: adding another requirement, such as the need to acquire, assess, adapt, and apply research evidence is viewed as a burden by many employees. However, there are numerous strategies within the seven main domains in the previously discussed framework that can address this issue. For example, under the domain of climate for research use, one of the sub-elements is instituting structures or positions accountable for encouraging research use in decision-making, which was one of the main elements identified by the participants in the study as an emerging development of supports for EIDM. Having one position in an organization that supports the use of EIDM can address this barrier. Studies have identified that long-term investments in human and/or institutional resources are a strength: and even if the investment is minimal i.e. just one individual, this can greatly support EIDM, since employees know who to turn to and who can support EIDM [33,37]. Another example to address this barrier could be within the main domain of ‘facilitating pull efforts’ , with the sub-element of enabling ‘easy access’ to the appropriate research through instituting physical tools to enable access. Accessing research is a barrier and can be time-consuming. Organizations should implement the necessary tools to enable their employees to quickly access the required research [38,39]. Ensuring appropriate journal subscriptions, providing relevant links on the organizations’ intranet, and access to one-stop shops with timely research are all examples of ways to enable easy access to research.

The final barrier, a negative attitude toward change, is a common barrier within the change management and KTE literature; implementing EIDM is a complex change and requires a process that will take time [40,41]. Numerous initiatives can be implemented to support employees through this change. For example, under the main domain of ‘pull efforts,’ two subdomains that could address this barrier are providing training and continuing education that include research use in decision-making [37] and conducting interactive workshops that focus on the use of research in decision-making [39]. By training and educating individuals as to (a) the importance of EIDM and (b) the practical execution of EIDM, individuals will not only understand the necessity of EIDM, but they will also learn how to acquire, assess, adapt, and apply research evidence so that the process will not be as overwhelming. Encouraging open discussions and workshops on EIDM will assist the individuals in slowly overcoming their negative attitude toward this change.

In addition to these examples that organizations can implement to address the specific barriers, the health system may want to explore a system approach to implementing some of the potential interventions. The interventions discussed can be costly or time consuming to develop; however, once they are developed or purchased, they may be relatively easy to transfer between organizations. For example, a health system can explore the possibility of bulk purchasing journal subscriptions and therefore, the barrier related to inadequate data access, as well as the future priority of facilitating pull (i.e., providing easy access to journals), can be addressed. This is an initiative that RHAs or larger organizations within a health system can pursue, yet smaller organizations can benefit from such interventions. Initiatives such as these can improve the implementation of EIDM.

Several strategies, based on the current research and existing literature, have been presented here to demonstrate some examples as to how to address the identified barriers. These are not mutually exclusive strategies: they need to be considered in the context of the organization and health system. Following the implementation of these strategies, their impact should be evaluated.
